# A Comparison of the Predictive Power of Anthropometric Indices for Hypertension and Hypotension Risk

**DOI:** 10.1371/journal.pone.0084897

**Published:** 2014-01-23

**Authors:** Bum Ju Lee, Jong Yeol Kim

**Affiliations:** Medical Research Division, Korea Institute of Oriental Medicine, Yuseong-gu, Deajeon, Republic of Korea; Daping Hospital, Third Military Medical University, China

## Abstract

**Background and Aims:**

It is commonly accepted that body fat distribution is associated with hypertension, but the strongest anthropometric indicator of the risk of hypertension is still controversial. Furthermore, no studies on the association of hypotension with anthropometric indices have been reported. The objectives of the present study were to determine the best predictors of hypertension and hypotension among various anthropometric indices and to assess the use of combined indices as a method of improving the predictive power in adult Korean women and men.

**Methods:**

For 12789 subjects 21–85 years of age, we assessed 41 anthropometric indices using statistical analyses and data mining techniques to determine their ability to discriminate between hypertension and normotension as well as between hypotension and normotension. We evaluated the predictive power of combined indices using two machine learning algorithms and two variable subset selection techniques.

**Results:**

The best indicator for predicting hypertension was rib circumference in both women (p = <0.0001; OR = 1.813; AUC = 0.669) and men (p = <0.0001; OR = 1.601; AUC = 0.627); for hypotension, the strongest predictor was chest circumference in women (p = <0.0001; OR = 0.541; AUC = 0.657) and neck circumference in men (p = <0.0001; OR = 0.522; AUC = 0.672). In experiments using combined indices, the areas under the receiver operating characteristic curves (AUC) for the prediction of hypertension risk in women and men were 0.721 and 0.652, respectively, according to the logistic regression with wrapper-based variable selection; for hypotension, the corresponding values were 0.675 in women and 0.737 in men, according to the naïve Bayes with wrapper-based variable selection.

**Conclusions:**

The best indicators of the risk of hypertension and the risk of hypotension may differ. The use of combined indices seems to slightly improve the predictive power for both hypertension and hypotension.

## Introduction

Along with the prevalence of obesity, high blood pressure is a common health issue in both women and men worldwide and has become a major disease in Korea [1]–[4]. Obesity is related to serious health problems such as hypertension [5], [6], ischemic stroke [7], cardiovascular disease [8]–[10], type 2 diabetes [5], [9], [11], metabolic syndrome [6], [12], sleep apnea [13], and dyslipidemia [6], [10]. Anthropometry is an easy, economical, effective, and reliable method that is useful as an initial screening tool for hypertension [14], [15]. Various anthropometric indices that describe obesity and body fat distribution have been developed; these include the body mass index (BMI), waist circumference (WC), the waist-to-hip ratio (WHR), and the waist-to-height ratio (WHtR).

Over the past few decades, the direct association between hypertension and anthropometric indices has been studied in many ethnic groups and countries, and attempts have been made to identify the best indicator of the risk of hypertension. However, the best predictor of hypertension (that is, the index with the strongest association with hypertension) differed among studies. For instance, several studies reported that the best single indicator of the risk of hypertension was BMI in Japanese women [16], Japanese men and women [17], and elderly Cuban women [18]. Some studies of Netherlands Antilles women and men [19], Italian men and women [20], Greek men and women [21], Taiwanese women [22], and Japanese men [16] suggested that WC was the strongest predictor. Meanwhile, other studies provided evidence that in elderly men in Barbados [18], Taiwanese men [22], and Korean men and women [23], WHtR was the best predictor of the risk of hypertension, whereas other studies demonstrated that WHR was the best predictor for Argentine men and women [24] and Australian indigenous men and women [25]. Some studies suggested that two or more indices, for example, BMI and WC in elderly Mexican men and women [15] and Croatian men and women [26] and BMI, WC, and WHtR in Brazilian men and women [14], were the best predictors. Therefore, among the anthropometric indices studied, the best predictor of the risk of hypertension remains controversial because of potential confounding factors including place of residence (rural or urban), ethnic group, gender, and economic status [2], [16], [18]. Furthermore, despite the fact that many studies of the association between hypertension and anthropometric indices have been performed, no studies have examined the association between hypotension and anthropometric indices or analyzed the predictive power of anthropometric indices for the risk of hypotension.

The purpose of the present study was to determine (1) which index is the strongest indicator for predicting the risk of hypertension and hypotension among various anthropometric indices in Korean people and (2) whether combined indices show better predictive power. To answer these questions, we examined the association of various anthropometric indices with hypertension and hypotension, assessed the predictive power of individual indices, and evaluated whether the use of combined indices can improve the predictive power for hypertension and hypotension compared with the use of individual indices. Unlike previous studies, which considered only a few indices as candidate predictors for the risk of hypertension, we considered a total of 41 indices measured in eight precise positions in the body or calculated as the ratio of the difference between two indices.

## Materials and Methods

### Data collection

This cross-sectional study examined the association between blood pressure status and anthropometric indices. All the data analyzed in the present study were obtained from the Korean Health and Genome Epidemiology study database (KHGES). Written informed consent was obtained from all participants, and the Korea Institute of Oriental Medicine (KIOM) Institutional Review Board approved the study.

The data for female and male groups were considered separately because human body shape differs according to gender. For the diagnosis of hypertension, the criteria of the World Health Organization [WHO] [27] were used. For the diagnosis of normotension, we used the criteria given in the 2013 European Society of Hypertension-European Society of Cardiology Guidelines for the management of arterial hypertension [28], the 2003 European Society of Hypertension-European Society of Cardiology guidelines for the management of arterial hypertension [29], and data from several previous studies [30]–[35]. The criteria of the National Heart, Lung, and Blood Institute [36] were used for the diagnosis of hypotension. In detail, the guidelines of reports [28] and [29] and previous studies [30]–[35] suggested that hypertension is defined as SBP ≥140 mmHg and/or DBP ≥90 mmHg, and normotension (precisely, Optimal, Normal and High normal) was defined as SBP <140 and/or DBP <90. The previous study [36] reported that hypotension was defined as SBP <90 and/or DBP <60. Therefore, in the present study, hypertension was defined as systolic blood pressure (SBP) ≥140 mmHg and/or diastolic blood pressure (DBP) ≥90 mmHg or physician-diagnosed hypertension. Normotension was defined as SBP ranging from 90–139 mmHg and DBP ranging from 60–89 mmHg, and hypotension was defined as SBP <90 mmHg and/or DBP <60 mmHg. If the participants had been diagnosed with hypertension in the past but were fully recovered, they were defined as normotensive participants. Ultimately, a total of 12789 subjects (7330 women and 5459 men) aged 21–85 years participated in this study. Of the women, 5627 were normotensive, 1703 had hypertension, and 344 had hypotension; of the men, 3777 were normotensive, 1580 had hypertension, and 102 had hypotension.

Demographic data for all of the subjects were documented. The anthropometric indices were measured by well-trained technicians or observers using standardized protocols with the subject in lightweight clothing without shoes. Body weight and height were measured to the nearest 0.1 kg and 0.1 cm, respectively (LG-150; G Tech International Co., Ltd., Uijeongbu), and body circumferences were measured using non-elastic tape in 8 detailed positions: forehead, neck, axilla, chest, rib, waist, pelvis, and hip. After measuring weight, height, and the circumferences of 8 specific areas, we computed BMI, the circumference ratios between 2 positions, and the ratios between waist circumference and height or weight [11], [37]. The baseline characteristics of the subjects and brief descriptions of the anthropometric indices used in the study are presented in Table 1.

**Table 1:** Baseline characteristics and brief descriptions of the anthropometric indices used in this study.

	Women			Men			
Index	Normotension	Hypertension	Hypotension	Normotension	Hypertension	Hypotension	Description
Subjects	5627	1703	344	3777	1580	102	Number of subjects
High BP	113.6 (11.51)	138.1 (15.55)	95.54 (11.33)	116.2 (10.68)	137.1 (14.31)	100.2 (11.71)	High blood pressure
Low BP	74 (7.355)	88.07 (9.882)	56.19 (4.611)	76.09 (7.054)	90.29 (9.639)	55.79 (4.457)	Low blood pressure
Height	156.3 (6.146)	154.4 (6.054)	156.7 (6.132)	168.5 (6.3208)	168.2 (6.385)	168.7 (8.015)	Height
Weight	57.49 (8.173)	59.52 (9.137)	53.86 (6.686)	68.25 (9.9321)	71.08 (10.95)	63.67 (10.26)	Weight
BMI	23.55 (3.28)	24.93 (3.413)	21.98 (2.754)	23.98 (2.9149)	25.07 (3.14)	22.31 (2.784)	Body mass index
Age	51.17 (13.79)	59.22 (11.46)	46.51 (15.81)	52.65 (14.05)	54.7 (12.82)	51.27 (19.63)	Age
ForeheadC	54.95 (1.731)	55.05 (1.782)	54.57 (1.648)	56.78 (1.737)	57.14 (1.888)	56.09 (1.566)	Forehead circumference
NeckC	33.04 (2.118)	34.18 (2.27)	32.14 (1.993)	37.46 (2.319)	38.49 (2.628)	36.06 (2.239)	Neck circumference
AxillaryC	87.16 (5.997)	90.1 (6.334)	83.98 (5.691)	95.14 (5.932)	97.43 (6.479)	92.68 (5.611)	Axillary circumference
ChestC	89.64 (7.727)	93.71 (8.107)	85.37 (7.13)	93.23 (6.246)	95.86 (6.848)	90.02 (6.4)	Chest circumference
RibC	78.66 (7.869)	83.52 (8.184)	75.04 (7.165)	87.03 (6.557)	90.11 (6.862)	83.63 (7.127)	Rib circumference
WaistC	82.95 (8.982)	87.94 (9.295)	78.49 (8.005)	86.17 (7.984)	89.7 (8.23)	82.04 (8.365)	Waist circumference (WC)
PelvicC	89.75 (7.095)	93.09 (7.435)	86.58 (6.653)	90.64 (6.145)	92.88 (6.718)	88.11 (6.494)	Pelvic circumference
HipC	92.61 (5.844)	94.46 (6.502)	90.55 (5.157)	93.17 (5.65)	94.96 (6.348)	91.75 (5.979)	Hip circumference
Forehead_Hip	0.595 (0.037)	0.585 (0.038)	0.604 (0.034)	0.611 (0.033)	0.604 (0.034)	0.614 (0.036)	Forehead-to-hip circumference ratio
Neck_Hip	0.357 (0.022)	0.363 (0.023)	0.356 (0.02)	0.403 (0.021)	0.406 (0.023)	0.394 (0.022)	Neck-to-hip circumference ratio
Axillary_Hip	0.942 (0.047)	0.955 (0.047)	0.928 (0.05)	1.022 (0.045)	1.027 (0.046)	1.011 (0.043)	Axillary-to-hip circumference ratio
Chest_Hip	0.968 (0.059)	0.992 (0.059)	0.943 (0.059)	1.001 (0.047)	1.01 (0.047)	0.982 (0.049)	Chest-to-hip circumference ratio
Rib_Hip	0.849 (0.063)	0.884 (0.061)	0.828 (0.06)	0.935 (0.052)	0.949 (0.048)	0.912 (0.056)	Rib-to-hip circumference ratio
Waist_Hip	0.895 (0.07)	0.931 (0.07)	0.866 (0.065)	0.924 (0.059)	0.944 (0.054)	0.894 (0.063)	Waist-to-hip circumference ratio (WHR)
Pelvic_Hip	0.969 (0.043)	0.986 (0.04)	0.956 (0.047)	0.973 (0.041)	0.978 (0.038)	0.961 (0.046)	Pelvic-to-hip circumference ratio
Forehead_Pelvic	0.616 (0.049)	0.595 (0.046)	0.634 (0.047)	0.629 (0.041)	0.618 (0.041)	0.64 (0.045)	Forehead-to-pelvic circumference ratio
Neck_Pelvic	0.369 (0.025)	0.368 (0.025)	0.372 (0.025)	0.414 (0.025)	0.415 (0.026)	0.41 (0.025)	Neck-to-pelvic circumference ratio
Axillary_Pelvic	0.973 (0.05)	0.97 (0.049)	0.972 (0.053)	1.051 (0.056)	1.051 (0.056)	1.055 (0.058)	Axillary-to-pelvic circumference ratio
Chest_Pelvic	1 (0.053)	1.007 (0.052)	0.987 (0.053)	1.03 (0.051)	1.033 (0.052)	1.023 (0.05)	Chest-to-pelvic circumference ratio
Rib_Pelvic	0.876 (0.054)	0.897 (0.053)	0.867 (0.057)	0.961 (0.05)	0.971 (0.049)	0.95 (0.052)	Rib-to-pelvic circumference ratio
Waist_Pelvic	0.923 (0.055)	0.944 (0.055)	0.906 (0.049)	0.95 (0.05)	0.965 (0.047)	0.93 (0.054)	Waist-to-pelvic circumference ratio
Forehead_Waist	0.67 (0.073)	0.633 (0.067)	0.702 (0.067)	0.664 (0.059)	0.642 (0.054)	0.69 (0.067)	Forehead-to-waist circumference ratio
Neck_Waist	0.401 (0.034)	0.391 (0.033)	0.412 (0.033)	0.437 (0.03)	0.431 (0.029)	0.443 (0.033)	Neck-to-waist circumference ratio
Axillary_Waist	1.057 (0.071)	1.03 (0.069)	1.075 (0.07)	1.109 (0.07)	1.09 (0.065)	1.136 (0.076)	Axillary-to-waist circumference ratio
Chest_Waist	1.085 (0.065)	1.069 (0.062)	1.092 (0.068)	1.086 (0.06)	1.072 (0.057)	1.102 (0.065)	Chest-to-waist circumference ratio
Rib_Waist	0.951 (0.054)	0.952 (0.05)	0.959 (0.059)	1.013 (0.05)	1.007 (0.047)	1.022 (0.052)	Rib-to-waist circumference ratio
Forehead_Rib	0.705 (0.07)	0.665 (0.064)	0.734 (0.066)	0.656 (0.047)	0.637 (0.045)	0.675 (0.056)	Forehead-to-rib circumference ratio
Neck_Rib	0.422 (0.031)	0.412 (0.03)	0.43 (0.029)	0.431 (0.024)	0.428 (0.024)	0.433 (0.027)	Neck-to-rib circumference ratio
Axillary_Rib	1.113 (0.061)	1.083 (0.058)	1.124 (0.061)	1.095 (0.051)	1.083 (0.048)	1.111 (0.056)	Axillary-to-rib circumference ratio
Chest_Rib	1.142 (0.052)	1.124 (0.05)	1.14 (0.055)	1.072 (0.039)	1.065 (0.038)	1.078 (0.04)	Chest-to-rib circumference ratio
Forehead_Chest	0.617 (0.053)	0.591 (0.05)	0.643 (0.05)	0.611 (0.037)	0.599 (0.038)	0.626 (0.043)	Forehead-to-chest circumference ratio
Neck_Chest	0.37 (0.024)	0.366 (0.025)	0.378 (0.024)	0.402 (0.02)	0.402 (0.023)	0.401 (0.024)	Neck-to-chest circumference ratio
Axillary_Chest	0.974 (0.038)	0.963 (0.037)	0.986 (0.036)	1.021 (0.026)	1.017 (0.028)	1.031 (0.033)	Axillary-to-chest circumference ratio
Forehead_Axillary	0.633 (0.043)	0.614 (0.041)	0.652 (0.041)	0.599 (0.033)	0.589 (0.035)	0.608 (0.034)	Forehead-to-axillary circumference ratio
Neck_Axillary	0.38 (0.02)	0.38 (0.021)	0.384 (0.02)	0.394 (0.02)	0.396 (0.022)	0.39 (0.02)	Neck-to-axillary circumference ratio
Forehead_Neck	1.669 (0.099)	1.616 (0.096)	1.703 (0.09)	1.52 (0.08)	1.49 (0.086)	1.56 (0.086)	Forehead-to-neck circumference ratio
WHtR	0.368 (0.05)	0.385 (0.054)	0.344 (0.041)	0.404 (0.052)	0.422 (0.057)	0.376 (0.052)	Waist-to-height circumference ratio

The data are expressed as the mean (standard deviation).

### Data analysis and experimental configuration

All the statistical analyses and the prediction experiments were conducted in SPSS 19 for Windows (SPSS Inc., Chicago, IL, USA) and Weka (the Waikato Environment for Knowledge Analysis data mining tool). In the statistical analyses of the individual indices, binary logistic regression (LR) was used to assess statistically significant differences between normotension and hypertension as well as between normotension and hypotension in both women and men after a standardization transformation was applied to the data. To analyze the predictive power of individual indices, binary logistic regression and naive Bayes algorithm (NB) were used to determine which index had better predictive power and produced more reliable results.

To compare the predictive power of combined indices, we used four prediction methods using two machine learning algorithms and two variable selection techniques to obtain more predictive power, to discover the optimal combined indices, to decrease the complexity of the model, and to produce more trustworthy results. Correlation-based feature subset selection (CFS) is a filter approach [38]; each of the two machine learning algorithms constructs prediction models using selected indices after the indices are selected once with the CFS technique. The wrapper-based approach employs specific machine learning algorithms in the variable selection process and attempts to find the optimal variable set for machine learning used in the experiment [39]. The four methods are as follows: naïve Bayes with CFS (NB-CFS method), logistic regression with CFS (LR-CFS method), naïve Bayes with wrapper (NB-wrapper method), and logistic regression with wrapper (LR-wrapper method). With regard to the search method, the greedy stepwise (backward search) method was used in all the experiments.

The area under the receiver operating characteristic curve (AUC) is generally used to assess discrimination power in the medical and biological fields. Therefore, we used the AUC as the primary criterion for the comparison of predictive power. In addition, we report the sensitivity, 1-specificity, and F-measure to provide a more detailed analysis of the prediction results for the combined indices. All the tests in the analysis of predictive power were performed using 10-fold cross validation.

## Results

### Comparison of hypertension and normotension

Table 2 presents the results of the statistical analysis and the comparison of the predictive power of individual anthropometric indices for hypertension and normotension. The best indicator of the risk of hypertension is RibC in both women (p = <0.0001; OR = 1.813; AUC = 0.669) and men (p = <0.0001; OR = 1.601; AUC = 0.627). Age is a more important predictor in women (p = <0.0001; OR = 1.931; AUC = 0.666) than in men (p = <0.0001; OR = 1.163; AUC = 0.532). In women, WaistC (called WC, p = <0.0001; OR = 1.728; AUC = 0.654), Rib_Hip (p = <0.0001; OR = 1.753; AUC = 0.656), and Forehead_Rib (p = <0.0001; OR = 0.536; AUC = 0.665) are useful predictors. In men, WaistC (p = <0.0001; OR = 1.560; AUC = 0.621), NeckC (p = <0.0001; OR = 1.527; AUC = 0.617), and Forehead_Rib (p = <0.0001; OR = 0.654; AUC = 0.614) are helpful indicators. Generally, when comparing hypertension with normotension, the predictive power and statistical significances of the anthropometric indices are better in women than in men.

**Table 2:** Statistical analysis of normotension vs. hypertension in women and men.

	Women				Men			
Index	P	OR	AUC (LR)	AUC (NB)	P	OR	AUC (LR)	AUC (NB)
Height	<0.0001	0.735	0.587	0.587	0.0569	0.944	0.514	0.507
Weight	<0.0001	1.264	0.567	0.568	<0.0001	1.314	0.575	0.574
BMI	<0.0001	1.494	0.621	0.621	<0.0001	1.437	0.601	0.6
Age	<0.0001	1.931	0.666	0.665	<0.0001	1.163	0.532	0.524
ForeheadC	0.0511	1.055	0.514	0.507	<0.0001	1.22	0.555	0.553
NeckC	<0.0001	1.676	0.645	0.645	<0.0001	1.527	0.617	0.617
AxillaryC	<0.0001	1.609	0.636	0.635	<0.0001	1.459	0.604	0.604
ChestC	<0.0001	1.67	0.646	0.646	<0.0001	1.504	0.61	0.609
RibC	<0.0001	1.813	0.669	0.669	<0.0001	1.601	0.627	0.627
WaistC	<0.0001	1.728	0.654	0.654	<0.0001	1.56	0.621	0.621
PelvicC	<0.0001	1.582	0.63	0.63	<0.0001	1.427	0.595	0.593
HipC	<0.0001	1.349	0.582	0.578	<0.0001	1.356	0.582	0.58
Forehead_Hip	<0.0001	0.753	0.578	0.576	<0.0001	0.796	0.563	0.561
Neck_Hip	<0.0001	1.264	0.566	0.562	<0.0001	1.167	0.547	0.544
Axillary_Hip	<0.0001	1.313	0.577	0.577	0.0003	1.116	0.532	0.528
Chest_Hip	<0.0001	1.512	0.617	0.615	<0.0001	1.21	0.553	0.555
Rib_Hip	<0.0001	1.753	0.656	0.655	<0.0001	1.342	0.581	0.581
Waist_Hip	<0.0001	1.669	0.641	0.641	<0.0001	1.42	0.597	0.597
Pelvic_Hip	<0.0001	1.507	0.608	0.605	<0.0001	1.137	0.528	0.522
Forehead_Pelvic	<0.0001	0.633	0.624	0.623	<0.0001	0.755	0.574	0.572
Neck_Pelvic	0.1672	0.962	0.507	0.503	0.1233	1.047	0.51	0.514
Axillary_Pelvic	0.0158	0.935	0.516	0.512	0.6892	0.988	0.498	0.478
Chest_Pelvic	<0.0001	1.159	0.545	0.544	0.0221	1.071	0.521	0.519
Rib_Pelvic	<0.0001	1.468	0.613	0.613	<0.0001	1.226	0.56	0.56
Waist_Pelvic	<0.0001	1.468	0.608	0.607	<0.0001	1.37	0.594	0.592
Forehead_Waist	<0.0001	0.569	0.65	0.65	<0.0001	0.66	0.611	0.609
Neck_Waist	<0.0001	0.741	0.585	0.584	<0.0001	0.813	0.549	0.549
Axillary_Waist	<0.0001	0.679	0.607	0.607	<0.0001	0.758	0.575	0.571
Chest_Waist	<0.0001	0.782	0.565	0.564	<0.0001	0.788	0.565	0.563
Rib_Waist	0.4669	1.02	0.503	0.524	0.0001	0.889	0.531	0.527
Forehead_Rib	<0.0001	0.536	0.665	0.664	<0.0001	0.654	0.614	0.612
Neck_Rib	<0.0001	0.699	0.598	0.594	<0.0001	0.869	0.533	0.528
Axillary_Rib	<0.0001	0.602	0.641	0.641	<0.0001	0.779	0.571	0.568
Chest_Rib	<0.0001	0.704	0.6	0.599	<0.0001	0.813	0.561	0.558
Forehead_Chest	<0.0001	0.593	0.642	0.642	<0.0001	0.703	0.597	0.597
Neck_Chest	<0.0001	0.854	0.542	0.541	0.5794	0.984	0.484	0.525
Axillary_Chest	<0.0001	0.737	0.588	0.588	<0.0001	0.857	0.544	0.543
Forehead_Axillary	<0.0001	0.621	0.63	0.629	<0.0001	0.732	0.588	0.585
Neck_Axillary	0.3817	1.024	0.503	0.501	0.0238	1.07	0.517	0.517
Forehead_Neck	<0.0001	0.577	0.65	0.649	<0.0001	0.687	0.609	0.608
WHtR	<0.0001	1.39	0.597	0.597	<0.0001	1.381	0.59	0.588

The AUC values were calculated using 10-fold cross validation. OR: odds ratios; AUC: area under the receiver operating characteristic curve; LR: logistic regression; NB: naïve Bayes.

### Comparison of hypotension and normotension

Table 3 lists the results of the statistical analysis and the predictive power of individual indices comparing hypotension with normotension. The strongest predictor in women is ChestC (p = <0.0001; OR = 0.541; AUC = 0.657); however, the best indicator in men is NeckC (p = <0.0001; OR = 0.522; AUC = 0.672). BMI, WaistC, and WHtR are useful indicators in both women (p = <0.0001; OR = 0.573; AUC = 0.64, p = <0.0001; OR = 0.582; AUC = 0.648, p = <0.0001; OR = 0.573; AUC = 0.639, respectively) and men (p = <0.0001; OR = 0.545; AUC = 0.654, p = <0.0001; OR = 0.593; AUC = 0.646, p = <0.0001; OR = 0.566; AUC = 0.645, respectively). In addition, AxillaryC is a beneficial indicator in women (p = <0.0001; OR = 0.56; AUC = 0.651), and Waist_Hip (called WHR) is a useful predictor in men (p = <0.0001; OR = 0.594; AUC = 0.647).

**Table 3:** Statistical analysis of normotension and hypotension in women and men.

	Women				Men			
Index	p	OR	AUC (LR)	AUC (NB)	P	OR	AUC (LR)	AUC (NB)
Height	0.2874	1.061	0.5	0.494	0.8001	1.026	0.448	0.556
Weight	<0.0001	0.602	0.629	0.624	<0.0001	0.61	0.627	0.626
BMI	<0.0001	0.573	0.64	0.637	<0.0001	0.545	0.654	0.653
Age	<0.0001	0.72	0.583	0.575	0.3340	0.909	0.489	0.685
ForeheadC	0.0001	0.803	0.561	0.558	0.0001	0.667	0.627	0.622
NeckC	<0.0001	0.625	0.629	0.628	<0.0001	0.522	0.672	0.669
AxillaryC	<0.0001	0.56	0.651	0.65	<0.0001	0.652	0.612	0.608
ChestC	<0.0001	0.541	0.657	0.657	<0.0001	0.585	0.634	0.633
RibC	<0.0001	0.596	0.629	0.628	<0.0001	0.589	0.636	0.634
WaistC	<0.0001	0.582	0.648	0.647	<0.0001	0.593	0.646	0.646
PelvicC	<0.0001	0.62	0.635	0.635	<0.0001	0.659	0.623	0.621
HipC	<0.0001	0.683	0.6	0.596	0.0125	0.774	0.568	0.558
Forehead_Hip	<0.0001	1.275	0.57	0.562	0.4004	1.087	0.507	0.476
Neck_Hip	0.1307	0.918	0.526	0.53	<0.0001	0.651	0.617	0.615
Axillary_Hip	<0.0001	0.74	0.589	0.586	0.0180	0.788	0.563	0.552
Chest_Hip	<0.0001	0.639	0.624	0.623	<0.0001	0.66	0.61	0.613
Rib_Hip	<0.0001	0.706	0.597	0.596	<0.0001	0.652	0.62	0.615
Waist_Hip	<0.0001	0.653	0.626	0.625	<0.0001	0.594	0.647	0.643
Pelvic_Hip	<0.0001	0.745	0.592	0.587	0.0030	0.76	0.561	0.54
Forehead_Pelvic	<0.0001	1.409	0.605	0.606	0.0106	1.274	0.57	0.566
Neck_Pelvic	0.0308	1.122	0.531	0.519	0.0920	0.838	0.535	0.514
Axillary_Pelvic	0.6595	0.976	0.495	0.508	0.5924	1.055	0.52	0.467
Chest_Pelvic	<0.0001	0.784	0.572	0.569	0.1966	0.876	0.508	0.47
Rib_Pelvic	0.0018	0.836	0.55	0.542	0.0234	0.793	0.544	0.529
Waist_Pelvic	<0.0001	0.722	0.595	0.599	0.0001	0.676	0.598	0.594
Forehead_Waist	<0.0001	1.524	0.632	0.629	<0.0001	1.505	0.62	0.617
Neck_Waist	<0.0001	1.349	0.594	0.588	0.0599	1.195	0.536	0.504
Axillary_Waist	<0.0001	1.29	0.575	0.572	0.0001	1.443	0.605	0.6
Chest_Waist	0.0674	1.106	0.528	0.514	0.0068	1.297	0.579	0.567
Rib_Waist	0.0089	1.152	0.533	0.532	0.0579	1.197	0.544	0.552
Forehead_Rib	<0.0001	1.496	0.612	0.611	<0.0001	1.458	0.603	0.603
Neck_Rib	<0.0001	1.282	0.569	0.562	0.6280	1.049	0.461	0.518
Axillary_Rib	0.0011	1.198	0.543	0.543	0.0016	1.33	0.58	0.572
Chest_Rib	0.5219	0.965	0.495	0.511	0.1224	1.158	0.546	0.53
Forehead_Chest	<0.0001	1.621	0.638	0.637	0.0001	1.444	0.595	0.581
Neck_Chest	<0.0001	1.353	0.592	0.587	0.6022	0.949	0.482	0.546
Axillary_Chest	<0.0001	1.329	0.585	0.583	0.0002	1.413	0.577	0.564
Forehead_Axillary	<0.0001	1.546	0.628	0.622	0.0084	1.292	0.578	0.569
Neck_Axillary	0.0003	1.209	0.559	0.553	0.0260	0.793	0.556	0.533
Forehead_Neck	<0.0001	1.409	0.602	0.603	<0.0001	1.63	0.633	0.628
WHtR	<0.0001	0.573	0.639	0.634	<0.0001	0.566	0.645	0.644

The AUC values were calculated using 10-fold cross validation. OR: odds ratios; AUC: area under the receiver operating characteristic curve; LR: logistic regression; NB: naïve Bayes.

Age has a lower ability to discriminate between hypotension and normotension than between hypertension and normotension. In men, the AUC values for age determined by NB and LR were very different (i.e., the AUC for age by NB is the highest in men), although there was not a statistically significant difference between the ages of the hypotensive and normotensive groups (p = 0.334; OR = 0.909). This phenomenon is due to the difference in the intrinsic characteristics of the NB and LR algorithms. Thus, we can consider the use of another machine learning algorithm to obtain a model with better predictive power or better classification accuracy that is more suitable for the features of the data sets that are used in the experiment.

### Predictive power of combined indices

**Figure 1:**
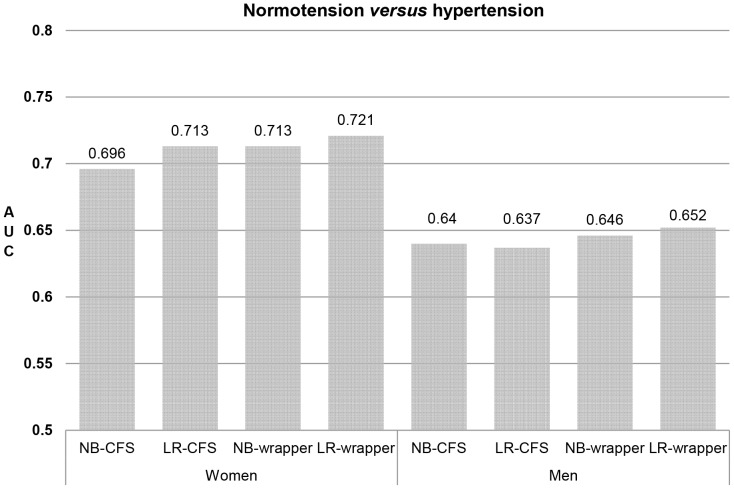
AUC values for normotension versus hypertension using combined indices in women and men. The AUC values were calculated using 10-fold cross validation. AUC: area under the receiver operating characteristic curve; LR: logistic regression; NB: naïve Bayes; CFS: correlation-based feature selection; wrapper: wrapper-based variable selection.

**Figure 2:**
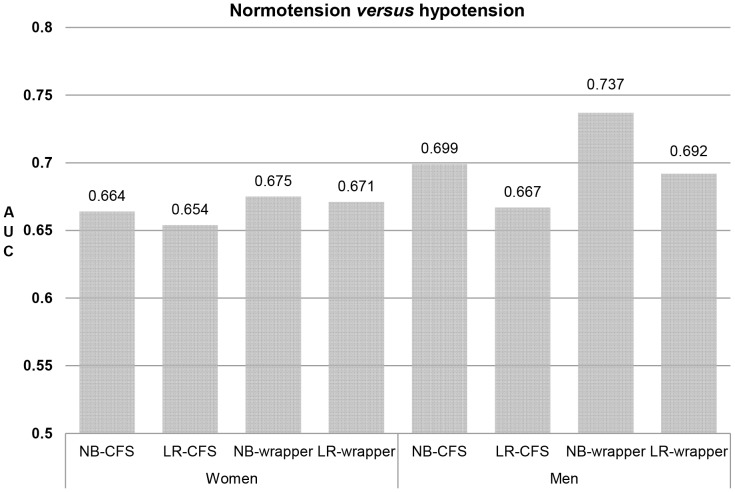
AUC values for normotension versus hypotension using combined indices in women and men. The AUC values were calculated using 10-fold cross validation. AUC: area under the receiver operating characteristic curve; LR: logistic regression; NB: naïve Bayes; CFS: correlation-based feature selection; wrapper: wrapper-based variable selection.

Figures 1 and 2 present the predictive power of combined indices for normotension versus hypertension and normotension versus hypotension. For predicting normotension versus hypertension, all four prediction methods had greater predictive power in women than in men, and the LR-wrapper method showed the highest predictive power in both women (AUC = 0.721) and men (AUC = 0.652). The models using combined indices showed slight improvements in the AUCs (0.052 in women and of 0.025 in men) compared with the single best predictor (i.e., RibC) among the individual indices.

For normotension versus hypotension, the predictive power of the indices was greater in men than in women, in contrast to the prediction of normotension versus hypertension. Of the four methods, the NB-wrapper method had the highest prediction performance in both women (AUC = 0.675) and men (AUC = 0.737). Compared with the best individual predictors (i.e., ChestC in women and NeckC in men), small improvements in the AUCs (approximately 0.018 in women and 0.065 in men) were observed in the models with combinations of indices.

Table 4 shows the detailed results of the prediction experiments using combined indices, including sensitivity, 1-specificity, and F-measure results. Table 5 lists the indices selected by the CFS and wrapper-based variable selection methods and the two machine learning algorithms. For instance, for predicting normotension versus hypertension, the model constructed using the LR-wrapper method for women included 12 indices (Height, Weight, BMI, Age, ChestC, Forehead_Hip, Waist_Hip, Chest_Pelvic, Waist_Pelvic, Axillary_Waist, Forehead_Rib, and Neck_Axillary) and showed that the sensitivities for normotension and hypertension were 0.969 and 0.109, respectively. The indices selected using the four methods differed according to the characteristics of the variable selection techniques and the two machine learning algorithms. Our results indicate that the model constructed by the LR-wrapper method was better than the other methods for predicting hypertension versus normotension in both men and women, whereas the model built with the NB-wrapper method was superior to the other methods for predicting hypotension versus normotension.

**Table 4:** Detailed results of prediction experiments using combined indices.

			Normotension vs. hypertension			Normotension vs. hypotension		
Gender	Method	Class	Sens.	1-spe.	F-mea.	Sens.	1-spe.	F-mea.
Women	NB-CFS	Normotension	0.726	0.452	0.78	0.78	0.576	0.86
		Hypertension	0.548	0.274	0.447	0.424	0.22	0.169
	LR-CFS	Normotension	0.967	0.908	0.863	1	1	0.97
		Hypertension	0.092	0.033	0.153	0	0	0
	NB-wrapper	Normotension	0.889	0.696	0.847	0.955	0.852	0.951
		Hypertension	0.304	0.111	0.363	0.148	0.045	0.157
	LR-wrapper	Normotension	0.969	0.891	0.866	1	1	0.97
		Hypertension	0.109	0.031	0.18	0	0	0
Men	NB-CFS	Normotension	0.704	0.521	0.733	0.949	0.853	0.963
		Hypertension	0.479	0.296	0.438	0.147	0.051	0.097
	LR-CFS	Normotension	0.968	0.915	0.824	1	1	0.987
		Hypertension	0.085	0.032	0.146	0	0	0
	NB-wrapper	Normotension	0.796	0.613	0.776	0.998	0.971	0.986
		Hypertension	0.387	0.204	0.413	0.029	0.002	0.053
	LR-wrapper	Normotension	0.964	0.885	0.826	1	1	0.987
		Hypertension	0.115	0.036	0.192	0	0	0

The results were calculated using 10-fold cross validation. LR: logistic regression; NB: naïve Bayes; CFS: correlation based feature selection; wrapper: wrapper-based variable selection; Sens.: sensitivity; 1-spe.: 1-specificity; F-mea.: F-measure.

**Table 5:** Selected indices based on the CFS and wrapper-based variable selection techniques.

Gender	Class	Method	Num.	Selected indices
Women	Normotension vs. Hypertension	CFS	15	Height, Age, NeckC, AxillaryC, RibC, WaistC, PelvicC, Rib_Hip, Waist_Hip, Pelvic_Hip, Rib_Pelvic, Axillary_Rib, Chest_Rib, Axillary_Chest, Forehead_Neck
		NB-wrapper	9	Height, Age, ForeheadC, NeckC, HipC, Axillary_Hip, Axillary_Pelvic, Chest_Pelvic, Chest_Rib
		LR-wrapper	12	Height, Weight, BMI, Age, ChestC, Forehead_Hip, Waist_Hip, Chest_Pelvic, Waist_Pelvic, Axillary_Waist, Forehead_Rib, Neck_Axillary
	Normotension vs. Hypotension	CFS	18	Weight, BMI, Age, ForeheadC, NeckC, AxillaryC, WaistC, PelvicC, Forehead_Hip, Axillary_Hip, Chest_Hip, Waist_Hip, Pelvic_Hip, Chest_Pelvic, Rib_Waist, Neck_Rib, Forehead_Chest, Axillary_Chest
		NB-wrapper	9	Weight, Age, ForeheadC, NeckC, AxillaryC, Chest_Hip, Pelvic_Hip, Waist_Pelvic, Rib_Waist
		LR-wrapper	12	Weight, Age, ForeheadC, Axillary_Hip, Rib_Hip, Axillary_Waist, Rib_Waist, Forehead_Rib, Neck_Rib, Chest_Rib, Forehead_Axillary, Neck_Axillary
Men	Normotension vs. Hypertension	CFS	18	Age, ForeheadC, NeckC, AxillaryC, ChestC, RibC, WaistC, PelvicC, HipC, Rib_Hip, Waist_Hip, Rib_Pelvic, Waist_Pelvic, Chest_Waist, Forehead_Rib, Chest_Rib, Axillary_Chest, Forehead_Neck
		NB-wrapper	15	Height, Age, ForeheadC, NeckC, AxillaryC, HipC, Rib_Hip, Pelvic_Hip, Neck_Pelvic, Waist_Pelvic, Chest_Waist, Chest_Rib, Neck_Chest, Axillary_Chest, Forehead_Neck
		LR-wrapper	19	Height, ForeheadC, NeckC, AxillaryC, RibC, PelvicC, Forehead_Hip, Chest_Hip, Rib_Hip, Pelvic_Hip, Forehead_Waist, Axillary_Waist, Rib_Waist, Neck_Rib, Axillary_Rib, Chest_Rib, Forehead_Axillary, Forehead_Neck, WHtR
	Normotension vs. Hypotension	CFS	10	Weight, Age, ForeheadC, NeckC, RibC, PelvicC, Neck_Hip, Waist_Hip, Pelvic_Hip, Waist_Pelvic
		NB-wrapper	7	Height, BMI, Age, ForeheadC, Neck_Hip, Axillary_Hip, Axillary_Chest
		LR-wrapper	13	BMI, ForeheadC, RibC, HipC, Forehead_Hip, Axillary_Pelvic, Rib_Pelvic, Waist_Pelvic, Rib_Waist, Axillary_Chest, Forehead_Axillary, Neck_Axillary, Forehead_Neck

## Discussion

Numerous epidemiologic studies support an association between obesity and blood pressure, and many studies have focused not only on the association between hypertension and anthropometric indices but also on identifying the best indicator of hypertension among anthropometric indices. The strongest association or the best predictor of the risk of hypertension can differ according to residence area (rural or urban) [2], gender [16], [18], cultural group [18], and country or ethnic group. Specifically, studies have stated that BMI is the strongest indicator of hypertension in Japanese women [16], Japanese men and women [17], elderly Cuban women [18], and Indian men and women [40]. For example, through analyses using AUC values and odds ratios, Gupta and Kapoor [40] documented that an elevated BMI was the strongest indicator of having hypertension in a North Indian population, while many studies have suggested that WC is the best predictor in elderly women from Barbados [18], elderly Cuban men [18], Netherlands Antilles women and men [19], Italian men and women [20], Brazilian women [41], Indian men and women [42], Greek men and women [21], Taiwanese women [22], and Japanese men [16]. In studies of elderly men from Barbados [18], Iraqi men and women [43], and Taiwanese men [22], WHtR was the strongest predictor of hypertension. For instance, Ashwell and colleagues [44] documented that the predictive power of WHtR for hypertension risk was better than that of BMI and WC in both men and women based on a meta-analysis of 18 studies of men and 19 studies of women from various countries. WHR is the best predictor in Argentine men and women [24] and Australian indigenous men and women [25]. For example, a study by Li and McDermott [25] suggested that BMI was the poorest predictor of hypertension in Australian indigenous men and women, whereas WHR was the strongest predictor among several indices. Other studies documented that both BMI and WC are the strongest indicators in elderly Mexican men and women [15] and Croatian men and women [26] and that BMI, WC, and WHtR are the best predictors in Brazilian men and women [14]. In Brazilian men and women [45] and in Chinese men and women in Hong Kong [46], both WHtR and WHR are the strongest predictors of the risk of hypertension. In Korea, hypertension is common and represents a major public health problem [3], [4], [23]. A number of studies have been performed to measure the association of hypertension with anthropometric indices and to determine the best predictor of the risk of hypertension in both women and men. A study by Kim and colleagues [3] reported that hypertension is associated with BMI, family history of hypertension, alcohol intake, and place of residence (rural or urban), and a study by Jo and colleagues [4] stated that BMI and WC are associated with hypertension in Korean men and women. Park and colleagues [23] suggested that WHtR is a better predictor of hypertension than WC and BMI. Our results differ from those of previous studies conducted in Korea. The reason for this is that previous studies only examined the association or predictive power of a few indices, whereas we used various indices measured in specific areas of the body (i.e., RibC, AxillaryC, and NeckC, the best predictors of hypertension or hypotension in our study, were not used in previous studies).

Jo and colleagues [4] showed that the association between age and hypertension is stronger in Korean women than in Korean men, although age was significantly associated with hypertension in both sexes. Our results agree with those of Jo and colleagues [4]; our data indicate that the association between age and hypertension is higher in women than in men and that the predictive power of age for hypertension is stronger in women than in men. Previously, the association of isolated systolic hypertension (ISH) with anthropometric indices in both Korean men and women was identified by two studies [1], [2] that stated that increasing age, BMI, and WHR are associated with ISH risk. However, one study suggested that geographical differences (urban versus rural area of residence) were not associated with ISH [1], whereas another study argued that the prevalence of ISH differed in rural and urban areas; thus, different results related to hypertension in rural and urban areas have been obtained [2]. Unfortunately, our study does not contribute to the understanding of geographical differences in hypertension because we did not consider residence area in our data analysis.

The results of several previous studies conducted in other countries corroborate our finding of an association of NeckC and ChestC with blood pressure. In studies on the association of NeckC with blood pressure [47]–[53], Ben-Noun and Laor [47], [48] reported that NeckC is positively correlated with diastolic and systolic blood pressure and that it is correlated with WaistC and Waist_Hip in Israeli men and women. The authors argued that changes in NeckC induce changes in diastolic and systolic blood pressure. In Chinese men and women [49], Zhou and colleagues demonstrated that NeckC is positively correlated with diastolic and systolic blood pressure, total cholesterol, and triglyceride levels and showed that it independently contributes to the identification of cardiometabolic syndrome beyond BMI, WaistC, and Waist_Hip indices, although the predictive power of NeckC was relatively lower than those of the other indices. They argued that NeckC is a more efficient and reliable anthropometric index than WaistC because WaistC is affected by stomach contents, respiratory movements, and clothing. In Argentine women and men [50], Alfie and colleagues presented evidence that neck obesity is associated with an increase in the prevalence of hypertension and showed that NeckC is a valuable index for discriminating hypertension and normotension. Furthermore, they indicated that NeckC was particularly helpful in distinguishing between hypertensive and normotensive individuals with normal abdominal measurements obtained by the WaistC index. In the Framingham Heart Study [51], Preis and colleagues studied the association of NeckC with cardiometabolic risk factors such as blood pressure, triglycerides, fasting plasma glucose, insulin, proinsulin, and cholesterol and showed that NeckC is positively associated with diastolic blood pressure in men and positively associated with systolic blood pressure in both men and women. In Brazilian women [52], Tibana and colleagues suggested that women with NeckC ≥35 cm had higher values of systolic blood pressure, glycated hemoglobin, and glucose compared with women with NeckC <35 cm and argued that women with higher NeckC tend to have higher cardiovascular risk factors. A study by Laakso and colleagues [53] tested the association of NeckC with WaistC, BMI, and Waist_Hip as well as with hypertension, lipid disorders, glucose intolerance, and hyperinsulinemia in Finnish men and women. The authors concluded that BMI, Waist_Hip, and WaistC correlated strongly with NeckC; the odds ratios for hypertension in the highest quintile of NeckC compared with the lowest quintile were 2.94 in women and 3.21 in men after adjustment of BMI in logistic regression.

In studies on the association of ChestC or breast circumference and blood pressure [54]–[57] in European women in the Netherlands, Poland, Sweden, and Italy [54], Seidell and colleagues documented that breast circumference and WaistC indices were positively correlated with diastolic blood pressure in women aged 38 years, while circumference ratios such as WHR, waist–to-thigh, and breast-to-hip were not associated with diastolic blood pressure. These authors suggested that the breast circumference index is strongly associated with metabolic risk indicated by diastolic blood pressure and by total cholesterol, HDL cholesterol, triglyceride, and insulin levels in women. In Chinese twins 7–12 years of age [55], Wang and colleagues found significant correlations of systolic and diastolic blood pressures with ChestC, weight, height, subscapular skin-fold and BMI and argued that blood pressure is less strongly associated with inherited factors than with environmental factors. Yao and colleagues [56] examined predictions of coronary heart disease mortality based on many anthropometric indices of white middle-aged railroad employees in the United States and found that systolic blood pressure was significantly correlated with ChestC and BMI. This study also indicated that the ratio of ChestC to biacromial diameter was significantly associated with coronary heart disease mortality. In Indian adults [57], Badaruddoza and Kumar studied the association of blood pressure with anthropometric indices in parent and child generations based on 1096 subjects comprising 350 families. The authors showed that ChestC, WaistC, arm circumference, calf circumference, weight, head length, biceps skinfold, triceps skinfold, and subscapular skinfold were significantly correlated with blood pressure in both parent and child generations; further, they found that the general household environment for several anthropometric measurements was one of the critical determinants of systolic and diastolic blood pressures and mean arterial blood pressure. However, despite the results of the present study and of other studies, we think that the reproducibility of chest, rib, and neck circumference measurements should be addressed and that additional studies on the possible association of neck, chest, and rib circumferences with blood pressure should be conducted in different countries and ethnic groups.

Although numerous studies of the possible association between blood pressure and anthropometric indices have been conducted in many countries and ethnic groups, no published studies have addressed possible anthropometric predictors of hypotension. Low diastolic pressure (i.e., hypotension) is a risk factor for dementia and Alzheimer's disease [58], [59]. Verghese and colleagues [58] stated that old people with continuously low blood pressure are at higher risk for dementia. Similar results supporting an association between low diastolic blood pressure or hypotension in later life with dementia and Alzheimer's disease were summarized in a recent review by Kennelly and colleagues [59]. Our findings show that many anthropometric indices such as BMI, NeckC, WHtR, and WaistC indices are associated with hypotension in Korean women and men; furthermore, analysis of the predictive power of the indices showed that the best predictors of hypotension and hypertension may differ.

Based on the odds ratios and p-values observed in this study, many of the individual indices that were measured showed statistically significant differences between individuals with hypertension or hypotension and those with normotension. However, it is difficult to achieve good predictive power for hypotension versus normotension in individual or combined indices because of the lack of normotensive samples in this study. In empirical applications in many research fields, a class imbalance problem (imbalanced data set) frequently occurs due to the presence of a large number of samples or subjects in one class and few samples in the other class [60]. For example, with regard to medical diagnoses, subjects with a given disease are usually rarer than normal subjects [61], [62]. The class imbalance problem is a significant drawback for prediction performance in most standard machine learning algorithms, including binary logistic regression, because most traditional classification algorithms are designed with equal sample sizes of negative and positive classes [63], [64] and are based on a hypothesis of a truly equivalent cost for incorrect predictions in each class for the maximal number of correct classified samples [62]. In reality, unusual and rare samples are notably more difficult to predict than typical and large samples [62], [65]. In our data, a problem of this nature arose. For instance, although the normotensive sample of men was very large (3777), there were very few men with hypotension in our sample (102). Therefore, as shown in Table 4, the sensitivity values for normotension showed good predictive power but the sensitivity values for hypotension were very poor. Several research fields, including data mining, statistics, medical science, and machine learning, have attempted to solve the class imbalance problem using under- and over-sampling, synthetic sampling, and cost-sensitive learning [65]. However, in the present study, we used original data that have characteristics typical of data in the epidemiological and medical fields; we did not apply any sampling methods and focused on the reliability of the predictive power of individual indices and combined indices using two machine learning algorithms and 10-fold cross-validation.

The limitations of the present study include the inability to make causal inferences about the associations between hypotension and hypertension and anthropometric indices because the study is cross-sectional. The nature of cross-sectional studies precludes conclusions about cause–effect relationships. In addition, the number of men with hypotension was very small compared with the number of men with hypertension, which may result in a class imbalance problem for classifications or predictions. Finally, although prediction methods that use several anthropometric indices slightly improve predictive power for blood pressure status, from a practical point of view these methods have several weaknesses, such as the increase of the number of body measurements needed and the increased time required for the calculation of the ratio of two anthropometric indices. As a result, methods that use combined anthropometric indices require more human resources, better control of the measured data, and additional time for measurement, calculation, and management.

## Conclusion

High blood pressure, which is a major health problem worldwide, is associated with adiposity and body fat distribution. Until now, studies have focused on the association between anthropometry and hypertension but not on the possible associations between anthropometric indicators and hypotension. In this study, we examined the association between anthropometric indicators and hypertension and hypotension in Korean adults and compared the predictive power of each individual index using binary logistic regression and naive Bayes. In addition, we used combined indices to achieve better predictive power with 4 prediction methods. Our findings suggest that the best predictors of hypertension and hypotension risk may differ. In both women and men, RibC seems to be the best predictor of hypertension, whereas the strongest indicators of hypotension appear to be ChestC in women and NeckC in men. The use of combined indices appears to slightly improve the predictive power of anthropometry for both hypertension and hypotension. Our findings provide additional information that may help develop a better initial screening tool for hypertension and hypotension. To our knowledge, this is the first report of an association between anthropometric indicators and hypotension that includes a comparison of the predictive power of various indices for hypertension and hypotension.
